# Rs12970134 near *MC4R* is associated with appetite and beverage intake in overweight and obese children: A family-based association study in Chinese population

**DOI:** 10.1371/journal.pone.0177983

**Published:** 2017-05-16

**Authors:** Shuo Wang, Jieyun Song, Yide Yang, Nitesh V. Chawla, Jun Ma, Haijun Wang

**Affiliations:** 1 Institute of Child and Adolescent Health of Peking University, School of Public Health, Peking University Health Science Center, Beijing, China; 2 Interdisciplinary Center for Network Science and Applications (iCeNSA), University of Notre Dame, Notre Dame, IN, United States of America; 3 Department of Computer Science and Engineering, University of Notre Dame, Notre Dame, IN, United States of America; Indiana University Richard M Fairbanks School of Public Health, UNITED STATES

## Abstract

**Background:**

Recent studies indicated that eating behaviors are under genetic influence, and the melanocortin 4 receptor (*MC4R*) gene polymorphisms can affect the total energy intake and the consumption of fat, protein and carbohydrates. Our study aims at investigating the association of the *MC4R* polymorphism with appetite and food intake among Chinese children.

**Methods:**

A family-based association study was conducted among 151 Chinese trios whose offsprings were overweight/obese children aged 9–15 years. The rs12970134 near *MC4R* was genotyped, and the Children Eating Behavior Questionnaire (CEBQ) and a self-designed questionnaire measuring food intake were performed. The FBAT and PBAT software packages were used.

**Results:**

The family-based association analysis showed that there was a significant association between rs12970134 and obesity (Z = 2.449, *P* = 0.014). After adjusting for age, gender and standardized BMI, rs12970134 was significantly associated with food responsiveness (FR) among children (β'_b_ = 0.077, *P*_b_ = 0.028), and with satiety responsiveness (SR) in trios (*P* = -0.026). The polymorphism was associated with beverage intake (β'_b_ = 0.331, *P*_b_ = 0.00016 in children; *P* = 0.043 in trios), but not significantly associated with vegetable, fruit or meat intake (*P*>0.050). We further found a significant mediation effect among the rs12970134, FR and beverage intake (b = 0.177, *P* = 0.047).

**Conclusions:**

Our study is the first to report that rs12970134 near *MC4R* was associated with appetite and beverage intake, and food responsiveness could mediate the effect of rs12970134 on beverage intake in overweight and obese Chinese children population. Further studies are needed to uncover the genetic basis for eating behaviors, which could lead to develop and implement effective interventional strategies early in life.

## Introduction

The prevalence of childhood obesity has experienced a continuous increase worldwide. As reported in 2013, 23·8% of boys and 22·6% of girls were overweight/obese in developed countries, and 12.9% of boys and 13.4% of girls were overweight/obese in developing countries [1]. Moreover, evidence has established that obesity could raise the risk of multiple co-morbid complications, including Type 2 diabetes, cardiovascular disease and several cancers [2, 3].

It has long been appreciated that obesity is highly heritable [4]. More recent studies indicated that eating behaviors are also under genetic influence [5]. Subsequent to the fat mass and obesity associated (FTO) gene [6], the melanocortin 4 receptor (*MC4R*) gene was the second gene validated by the genome-wide association study (GWAS) for obesity [7]. In total, four variants annotated as *MC4R* polymorphisms (all located downstream of the *MC4R* gene) have been found to be associated with obesity or BMI, including rs12970134 [8,9], rs17782313 [7,10,11], rs571312 [12] and rs17700144 [13]. Out of the 4 variants, rs17700144 had the frequency of 0% in the Chinese Han Beijing population (Hapmap CHB database), while rs12970134 was closest to the *MC4R* gene and was in strong linkage disequilibrium (LD) with rs17782313 (D’ = 0.909, r2 = 0.826 in CHB) and rs571312 (D’ = 0.909, r2 = 0.826 in CHB), which could be used as the proxy of the other two polymorphisms. Polymorphisms near *MC4R* not only play an important role in the predisposition to obesity, but also affect eating behaviors directly. It was reported that the risk alleles of polymorphisms near *MC4R* were associated with higher total energy intake [14], and could increase the consumption of fat [14], protein [14] and carbohydrates [15]. Beside of food intake, the polymorphisms near *MC4R* were also found to influence appetite, measured by Children Eating Behavior Questionnaire (CEBQ). Two studies have used CEBQ to evaluate the effect of polymorphisms near *MC4R* on appetite [16,17], both conducted among Chilean children, indicating that the CC genotype carriers had a relatively higher CEBQ score of “enjoyment of food” subscale (*P* = 0.04) and a lower score of “satiety responsiveness” subscale (*P* = 0.02), as compared to children with TT genotype [17].

Previous studies focusing on the polymorphisms near *MC4R* and eating behaviors mostly relied on the case-control study design [14,15,17]. In 2010 Valladares, et al. collected 134 case-parent trios, but family-based analyses were only used to analyze the association of the polymorphisms near *MC4R* with obesity, but not the associations with eating behaviors [16]. The family-based association studies could examine associations within family groups, and so are robust to deal with population stratification, which is a known confounder of case–control studies [18]. Besides of the original qualitative TDT procedure, different methods and software packages have been designed for quantitative variables [19–22], and have been used to provide more credible evidence in terms of genetic polymorphisms and phenotypes [23–25].

Although studies have unraveled that the polymorphisms near *MC4R* could affect different aspects of eating behaviors, there was no evidence on their association with beverage intake, and no studies have reported their association with appetite among Chinese children. Our hypothesis was that rs12970134 near *MC4R* could affect appetite and further influence the food intake in overweight/obese Chinese children. Therefore, we conducted a family-based association study among 151 Chinese trios whose offsprings were overweight/obese children. The objectives of this study were: 1) To investigate the association between rs12970134 and appetite measured by CEBQ; 2) To identify the association between rs12970134 and beverage intake; and 3) To explore whether the appetite plays a mediator role between rs12970134 and beverage intake in overweight/obese Chinese children.

## Methods

### Subjects

A family-based association study was conducted among 151 Chinese parent-offspring trio families (453 samples in total), whose offsprings were overweight or obese children aged 9–15 years. Each trio consisted of 3 individuals: the father, mother and child. The study subjects came from the Nuclear Family Study of Childhood Obesity (NFSCO), recruited from 7 elementary schools and 3 middle schools in Chaoyang District and Changping District (both urban regions) of Beijing, China. We followed a family-based study design which was a special design for transmission/disequilibrium test (TDT). Family-based studies had the advantages of avoiding the influence of population substructure [26]. The uniform Body Mass Index (BMI) percentile criteria were used to determine overweight and obesity, which were derived in a representative Chinese population [27] and used in previous studies [28,29]. According to the criteria, the children and adolescents with an age- and gender-specific BMI ≥ 95^th^ percentile were defined as obese, whereas those with BMI between 85^th^ and 95^th^ percentile were defined as overweight. All the individuals were recruited with their voluntary participation. Written informed consent was provided by all participants. The study was approved by the Ethic Committee of Peking University Health Science Center.

### Measurements

Fasting venous blood samples were taken for DNA extraction. Parents’ age, gender, height and weight were reported by themselves and the children’s were reported by their parents. BMI was calculated accordingly. It has been shown that self-reported figural scales had a high predictive ability for identifying overweight/obese subjects, and can be used with confidence as proxies of BMI [30]. The sex- and age-specific BMI standard deviation score (BMI-sds) was calculated by using the growth reference data of the World Health Organization for children and adolescents aged 5–19 years (http://www.who.int/childgrowth/standards/bmi_for_age/en/).

Children’s appetite was evaluated using the Children’s Eating Behavior Questionnaire (CEBQ) completed by their parents. This questionnaire was first designed in 2001 and consisted of 35 items measuring 8 dimensions of eating behavior, and used a 5-point Likert-type scale [31]. The questionnaire developers conducted factor analyses and further recommended in 2007 that SR (satiety responsiveness) and SE (Slowness in Eating) should be merged into one scale to represent the satiety responsiveness [32]. Our research focused on three scales related to appetite: FR (food responsiveness, 5 items), SR (9 items, including 5 items for the original SR and 4 items for SE) and EF (enjoyment of food, 4 items). FR reflects the child’s interest in eating and the desire to spend time eating; SR reflects the degree to which children are capable of ceasing consumption in response to internal cues; and EF reflects the amount of enjoyment the child experiences while eating. Higher scores of FR and EF and lower scores of SR reflect higher levels of appetite. These three scales have demonstrated high internal consistency, good correspondence with observed behavioral measures of child appetite, and consistent associations with child weight [33], and the CEBQ questionnaire has been validated in a variety of different populations [34–36], including Chinese children population [37].

Food intake including vegetable intake, fruit intake, meat intake and beverage intake was investigated using a self-designed questionnaire and completed by the subjects themselves. This questionnaire was designed according to Block Kids Food Screener (BKFS), which was developed to evaluate dietary intake of nutrients and food groups in youth aged 2–17 years by NutritionQuest (Berkeley, CA, USA) in 2007 [38], and later validated by Hunsberger, et al. [39] in 2015 to be a useful dietary assessment instrument for the nutrients and food groups in children. For each category of food, children were asked to report the frequency and quantity of food consumed during the previous week, and then the number of days per week when consuming the specific category of food were multiplied by daily average servings in order to calculate the food intake per week.

### SNP genotyping

The rs12970134 polymorphism was genotyped using genomic DNAs extracted from blood leukocytes by the phenol-chloroform extraction method. Genotyping was conducted on MassARRAY System (Sequenom, San Diego, CA, USA). Primers, including a pair of amplification primers and an extension primer, were designed with Sequenom MassArray Assay Design Suite. A multiplex polymerase chain reaction was performed, and unincorporated double stranded nucleotide triphosphate bases were dephosphorylated with shrimp alkaline phosphatase followed by primer extension. The purified primer extension reaction was spotted on to a 384-element silicon chip (SpectroCHIP, Sequenom) and analyzed in the Matrix assisted laser desorption ionization time of flight mass Spectrometry (MALDI-TOF MS, Sequenom). The resulting spectra were processed with MassArray Typer (Sequenom) (www.sequenom.com). The genotyping call rate of rs12970134 was 99.56%. All the experiments were done by investigators who were blind to the phenotypes.

### Statistical analyses

Quality control (QC) of the genotype was performed prior to association analysis by testing Mendelian inheritance consistency and Hardy-Weinberg equilibrium proportions. Regular statistical analyses were performed using SPSS (Statistical Package for the Social Sciences) version 18.0 software (SPSS, Chicago, IL, USA). Descriptive values were given as mean ± standard deviation or percentages, and the statistical differences in demographic and dietary characteristics between obese and overweight subjects were tested with t-tests (continuous variables) or Pearson Chi-square tests (categorical variables). Family-based associations between obesity and rs12970134 were tested in the family sample using family-based association tests [19] (FBAT version 2.0.4, www.biostat.harvard.edu/fbat/fbat.htm). Linear regression analyses were used to analyze the association between rs12970134 and dietary variables within the offsprings. Multiple approaches have been developed and different types of phenotypes could be analyzed including dichotomous (affection status), quantitative and censored (e.g., the age of onset) [40]. For the analyses of quantitative variables in trios, PBAT software package [20] was used. As compared to other computer programs that are available for family-based association tests, including FBAT, QTDT [21] and PDT [22], PBAT was considered to have the advantage of handling various trait types, could include covariates and gene/covariate-interactions, and *P* values could be computed on the basis of both asymptotic theory and permutation tests [20]. We used the PBAT implementations for Windows XP that are freely available online (PBAT Version 3.61, www.hsph.harvard.edu/clange/default.htm). For both linear regression method and PBAT method, covariates of age, gender and BMI-sds were adjusted for stepwise. Quanto software (University of Southern California, Los Angeles, CA) was used to conduct power analysis. The polymorphism was analyzed under the additive model. A two-sided *P*<0.05 was considered as nominally significant.

The mediation analysis was based on the model brought forward by Baron, et al. [41]. According to the model, three multivariate linear regression models were constructed, all adjusting for age, gender and BMI-sds. In order to distinguish the equations, the independent variable, dependent variable and mediator were represented as *X*, *Y* and *M*, and the coefficients in the model were represented b*y a*, *b*, *c and c’*. *T*he first regression model used *c* as the coefficient of *X* in association with *Y*; the second model used *a* as the coefficient of *X* in association with *M*; the third model put *M* and *X* as independent variables simultaneously, and used *b* and *c’* as the coefficients in association with *Y*.

## Results

### Demographic and the dietary characteristics of the study subjects

The descriptive information on age, gender and anthropometrics is provided in Table 1. The parents had the age range of 32–55 years and 32–50 years, and the average of BMI of 26.3 kg/m^2^ and 25.0 kg/m^2^, for fathers and mothers respectively. The 151 recruited children aged 9–15 years with the average age of 11.7 years, and 68.2% of them were boys. Among them, 98 children were obese and 53 were overweight, and the average BMI of the offsprings was 26.6 kg/m^2^. We observed no difference between overweight and obese children in age, gender or height (*P*>0.05).

**Table 1:** Demographics and the dietary characteristics of the study subjects.

Variables	Father (n = 151)	Mother (n = 151)	Offspring (n = 151)			
			Total	Overweight (n = 53)	Obese (n = 98)	*P*
Age (years)	41.1±4.0	39.3±3.9	11.7±1.5	11.8±1.6	11.6±1.6	0.461
Males (n, %)	—	—	103, 68.2	32, 60.4	71, 72.4	0.128
Height (cm)	172.8±5.1	161.2±5.1	160.3±11.3	159.7±9.7	160.6±12.0	0.653
Weight (kg)	78.6±10.9	65.0±9.3	68.8±16.3	59.0±10.1	74.1±16.6	**1.2E-8**
BMI (kg/m^2^)	26.3±3.5	25.0±3.5	26.6±4.4	22.9±1.7	28.5±4.2	**1.1E-16**
FR	—	—	2.6±0.8	2.4±0.7	2.7±0.9	**0.035**
SR	—	—	2.3±0.5	2.3±0.5	2.3±0.5	0.664
EF	—	—	3.6±0.8	3.5±0.6	3.7±0.9	**0.030**
Vegetable intake*	—	—	10.0±5.6	8.4±4.9	10.7±5.8	**0.026**
Fruit intake*	—	—	9.2±5.8	8.3±5.4	9.6±6.0	0.268
Meat intake*	—	—	5.8±4.9	4.7±3.6	6.2±5.4	0.057
Beverage intake*	—	—	4.7±6.0	2.7±4.0	5.5±6.6	**0.003**

Values are provided as Mean±SD if not indicated otherwise.

*P* values < 0.05 are shown in bold.

BMI: body mass index; FR: Food responsiveness; SR: Satiety responsiveness; EF: Enjoyment of food.

* Different kinds of food intake were calculated in servings per week.

Table 1 also shows the appetite and food intake characteristics of the offsprings. Overall, obese children had significantly higher scores of appetite in food responsiveness and food enjoyment (2.7±0.9 vs 2.4±0.7, *P* = 0.035; 3.7±0.9 vs 3.5±0.6, *P* = 0.030), but no significant difference was found in satiety responsiveness (*P* = 0.664). In terms of food intake, the vegetable intake and the beverage intake among obese children were significantly higher than that of overweight children (10.7±5.8 vs 8.4±4.9, *P* = 0.026; 5.5±6.6 vs 2.7±4.0, *P* = 0.003). Obese children also reported slightly more meat intake and fruit intake than overweight children, but no significant differences were observed (*P*>0.05).

### Association between the rs12970134 polymorphism and obesity

The QC analyses found no discrepancies in Mendelian inheritance. The risk allele (A allele) frequency of rs12970134 polymorphism was 21.2% in children and 19.2% in parents, and the genotype distribution were in Hardy-Weinberg equilibrium both among children (*P* = 0.175) and among parents (*P* = 0.267).

We first conducted FBAT analyses using the 151 overweight and obese children as probands, no significant association was found between rs12970134 and ‘overweight + obesity’ (76 informative families, Z = 1.410, *P* = 0.159). However, we then used only obese children (N = 98) as probands, and FBAT analyses showed that there was significant positive association between rs12970134 and obesity (49 informative families, Z = 2.449, *P* = 0.014), revealing that the risk allele (A allele) of rs12970134 could increase the risk of childhood obesity. Further, we tested the continuous variable BMI-sds, but the association between rs12970134 and BMI-sds was not significant by PBAT analyses among 151 trios (*P* = 0.790), or by linear regression among 151 children (β' = 0.119, *P* = 0.147).

### Association between the rs12970134 polymorphism and appetite

Three scales of the CEBQ questionnaire were used to represent childhood appetite: food responsiveness, satiety responsiveness and enjoyment of food. To analyze the association between rs12970134 and each scale, we first constructed linear regression models among the children (a, b columns, as presented in Table 2), and then conducted PBAT analyses among the trios (c, d). For both methods, covariates were adjusted for as followings: first adjusted for age and gender (a, c) and then BMI-sds was additionally adjusted for (b, d). For PBAT analyses, the number of informative families was 76 among the 151 trios.

**Table 2:** Association between the *MC4R* rs12970134 polymorphism and appetite.

Variables	Means±SD			β'_a_	*P*_a_	β'_b_	*P*_b_	*P*_c_	*P*_d_
	GG(n = 91)	AG(n = 56)	AA(n = 4)						
FR	2.44±0.70	2.66±0.89	3.35±1.25	0.196	**0.017**	0.177	**0.028**	0.176	0.265
SR	2.37±0.50	2.28±0.57	1.78±0.27	-0.148	0.072	-0.153	0.064	-0.030*	-0.026*
EF	3.61±0.77	3.62±0.84	4.06±0.83	0.031	0.699	0.021	0.792	-0.885*	-0.760*

a: Standerdized β and *P* values of rs12970134 in children using Linear regression models adjusted for age and gender; b: In children adjusted for age, gender and BMI-sds; c: PBAT *P* values of rs12970134 in trios adjusted for age and gender. d: In trios adjusted for age, gender and BMI-sds.

* Negative *P* values were used in PBAT output for negative association.

*P* values < 0.05 are shown in bold.

*MC4R*: Melanocortin-4 receptor; FR: Food responsiveness; SR: Satiety responsiveness; EF: Enjoyment of food.

The association between rs12970134 and FR score was significant among children, both with BMI-sds as a covariate (β'_b_ = 0.077, *P*_b_ = 0.028) and without (β'_a_ = 0.196, *P*_a_ = 0.017), indicating that the rs12970134 A allele increased children’s food responsiveness level, but the result was not validated in PBAT analyses (*P*>0.05). There was marginal negative association between rs12970134 and SR score among children (β'_b_ = -0.153, *P*_b_ = 0.064), and the association was significant using PBAT analyses (*P* = -0.026), revealing that children with the rs12970134 A allele could be hard to have satiety responsiveness. EF was not significantly associated with rs12970134 in any of the analyses (*P*>0.05).

### Association between the rs12970134 polymorphism and food intake

Four aspects of food intake were analyzed: vegetable, fruit, meat and beverage intake. As illustrated in Table 3, out of the four aspects, only beverage intake was found significantly associated with rs12970134, both in children (β'_b_ = 0.331, *P*_b_ = 0.00016) and in trios PBAT analysis (*P* = 0.043), indicating that the A allele of rs12970134 could increase the beverage intake among overweight and obese children.

**Table 3:** Association between the MC4R rs12970134 polymorphism and food intake.

Variables	Means±SD			β'_a_	*P*_a_	β'_b_	*P*_b_	*P*_c_	*P*_d_
	GG(n = 91)	AG(n = 56)	AA(n = 4)						
Vegetable	9.64±5.42	10.68±6.01	10.50±4.95	0.088	0.337	0.063	0.482	0.434	0.438
Fruit	9.12±5.74	9.30±6.11	8.50±2.12	0.001	0.991	-0.004	0.963	-0.797*	-0.792*
Meat	5.55±4.20	5.67±5.53	17.50±4.95	0.124	0.163	0.123	0.170	0.761	0.763
Beverage	3.31±4.33	6.50±7.46	16.75±6.72	0.337	**0.00011**	0.331	**0.00016**	**0.039**	**0.043**

a: Standerdized β and *P* values of rs12970134 in children using Linear regression models adjusted for age and gender; b: In children adjusted for age, gender and BMI-sds; c: PBAT *P* values of rs12970134 in trios adjusted for age and gender. d: In trios adjusted for age, gender and BMI-sds.

* Negative *P* values were used in PBAT output for negative association.

Coefficients with *P*<0.05 are shown in bold.

MC4R: Melanocortin-4 receptor; FR: Food responsiveness; SR: Satiety responsiveness; EF: Enjoyment of food.

### Mediation analysis of the rs12970134 polymorphism, food responsiveness and beverage intake

**Fig 1:**
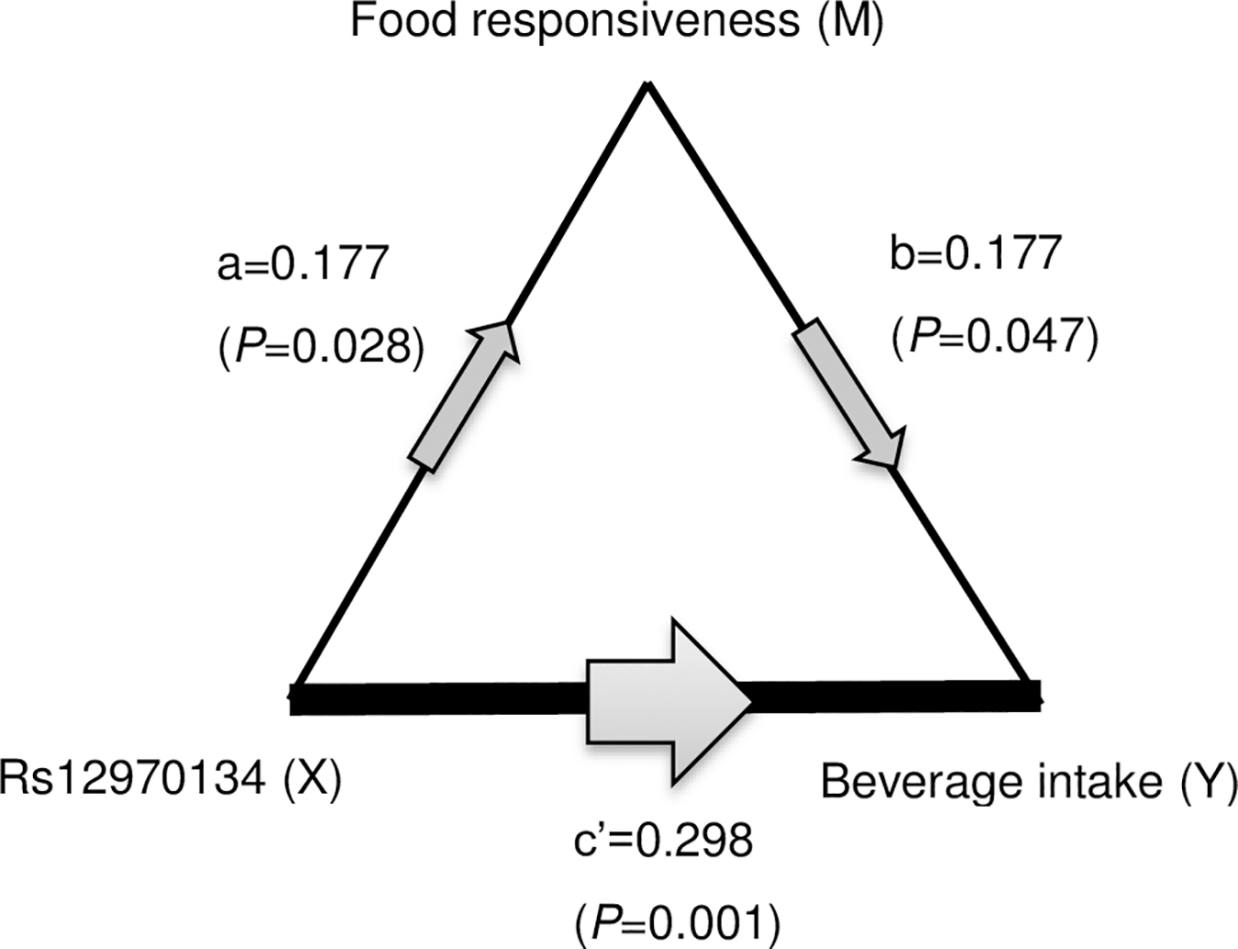
Mediation analysis of rs12970134, food responsiveness and beverage intake among overweight and obese children.

Mediation analysis was conducted among 151 children. Since rs12970134 was associated with both FR and beverage intake, we used a mediation model to examine whether rs1297134 could mediate the effect of rs12970134 and beverage intake. Fig 1 illustrates the mediation model among the three variables, with several multivariate linear regression models adjusted for age, gender and BMI-sds, as described in Methods: (1) Y = cX + p_1_age + q_1_gender + r_1_ BMI-sds + e_1_; (2) M = ax + p_2_age+ q_2_gender + r_2_ BMI-sds + e_2_; (3) Y = c’X + bM + p_3_age+ q_3_gender + r_3_ BMI-sds + e_3_. Significant mediation effect of FR was found on the association between rs12970134 and beverage intake (b = 0.177, *P* = 0.047), although the mediation effect was incomplete (rs12970134 could still affect beverage intake significantly after adjusting for FR, c’ = 0.298, *P* = 0.001). Further calculation (ab/c × 100%) reflected that the mediation effect of FR could explain 9.5% of the total association between rs12970134 and beverage intake.

## Discussion

In this study, we conducted a family-based association study among 151 Chinese trios whose offsprings were overweight/obese children aged 9–15 years. We validated the association between the polymorphism rs12970134 near *MC4R* and obesity, and found its association with childhood appetite (food responsiveness and satiety responsiveness) and beverage intake in overweight/obese children. To our knowledge, this study is the first to report that the *MC4R* rs12970134 polymorphism was associated with appetite and beverage intake, and food responsiveness could mediate the effect of rs12970134 on beverage intake in overweight and obese Chinese population.

*MC4R* gene is located on chromosome 18q22 and encodes for the melanocortin 4 receptor, which is a G protein-coupled receptor and is critically involved in energy balance regulation [42]. The expression of *MC4R* is in multiple regions of the brain including thalamus and hypothalamus, and can influence the leptin-melanocortin signaling system [43]. *MC4R* expressed in the paraventricular nucleus has a critical role in the mechanisms of food intake [44], and the *MC4R* knockout mouse demonstrated the characteristics of being obesity, hyperphagia, and hyperinsulinemia [45]. Genotype-Tissue Expression Project (GTEx) showed that there were 146 eQTLs in the gene *MC4R*, with the majority (145 variants) of them differentially expressed in brain, and 1 variant differentially expressed in testis. It was reported by Yoon, et al. that there was significant coexpression of *MC4R* and the dopamine D2 receptor in the hypothalamic area, and also in the bed nucleus of the stria terminalis (BNST) area which was an important site for food reward [46].

An important point should be noted that the rs12970134 polymorphism was not located in the *MC4R* gene but was an intergenic variant located in-between the gene *MC4R* and *LOC342784*. For a long time, rs12970134 was referred as an ‘*MC4R* variant/polymorphism’ or a ‘variant/polymorphism near *MC4R*’, but the link between rs12970134 with the gene *LOC342784* was ignored. We used the online tool SCAN (SNP and CNV Annotation Database, http://www.scandb.org/newinterface/about.html) to retrieve genes with variants in LD with rs12970134, and found that rs12970134 was not in LD with *MC4R*, but in strong LD with the gene *LOC342784* (r^2^ = 1). Actually, other polymorphisms near *MC4R* had the same situation. Rs12970134, rs17782313 and rs571312 were in complete LD with *LOC342784* (r^2^ = 1), rs17700144 was in strong LD with *LOC342784* (r^2^ = 0.867), and all the four SNPs were not in LD with *MC4R*. The gene *LOC342784* was a pseudo gene and currently we had no clues about its function, or whether or not it had function. Future genotype-phenotype association studies should be cautious about the SNP location and linkage in/near *MC4R* and *LOC342784*, and future functional researches should pay more attention on the gene *LOC342784*.

Rs12970134 was reported to be associated with obesity and obesity-related traits. In 2008, rs12970134 was reported by a GWAS to be significantly associated with waist circumference (*P* = 1.7×10^−9^), with homozygotes for the risk allele of rs12970134 have 2 cm increased waist circumference [8]. In 2009, another GWAS conducted by Thorleifsson, et al. [9] identified that rs12970134 A allele could increase the risk of obesity (OR 1.12, 95%CI 1.06–1.17, P = 9.9×10^−6^), and was positively associated with BMI (β = 4.38, P = 1.2×10^−12^) and weight (β = 4.68, *P* = 3.6×10^−13^). In this study, the association between rs12970134 and obesity was studied in a Chinese family-based association analysis. As to the allele frequency, the rs12970134 risk allele (A allele) frequency was 0.2119 in the current study, which was similar as compared to 0.2195 in the CHB population.

In our study the SNP was associated with obesity but not with ‘overweight + obese’. Although overweight and obesity are both indicators of adiposity, obesity actually represents a relatively extreme status while overweight represents the status in-between adiposity and normal weight. Combining overweight and obese children could weaken the effect of obesity-related SNP, resulting in the insignificance in a relatively small sample size. Besides, the association between rs12970134 and BMI-sds was not significant. It was because our study did not include normal-weight children, and the variation of BMI-sds was much smaller as compared to the general population.

We found no published literatures reporting the association between *MC4R* polymorphisms and appetite in Chinese population. There are several studies conducted among other ethnic populations but the results were inconsistent. Among them, two studies used the CEBQ questionnaire, both conducted among Chilean children. Valladares, et al. [16] first reported in 2010 that rs17782313 was negatively associated with satiety responsiveness subscale (combined average of satiety responsiveness and slowness in eating) (*P* = 0.01) and positively associated with enjoyment of food subscale (*P* = 0.03). Besides, there was a marginal association of 17782313 with food responsiveness subscale (*P* = 0.09). Vega, et al. reported that rs17782313 was associated with higher scores of uncontrolled eating in Chilean adults [47]. Obregón AM, et al. reported that rs17782313 was associated with food responsiveness (*P* = 0.02) [48]. Ho-Urriola, et al. later conducted a case-control study in 2014 [17] where 6 children with rs17782313 CC genotype and 60 children with TT genotype were matched by gender, age and BMI, and found that as compared to children with TT genotype, the CC genotype carriers had a relatively higher CEBQ score of “enjoyment of food” (*P* = 0.04) and a lower score of “satiety responsiveness” (*P* = 0.02). Our result on “satiety responsiveness” was in consistency with previous studies, but we did not find significant association between the polymorphism near *MC4R* and the “enjoyment of food” subscale. Another instrument to measure eating behaviors regarding cognition and motivation is the Three Factors Eating Questionnaire (TFEQ), which contains fifty-one items and three dimensions (factors) including disinhibition, restraint and hunger [49]. Stutzmann et al. conducted a study in 2438 18-89y French adults with obesity using the TFEQ, and found that thers17782313 C allele was positively associated with the score of hunger (*P* = 0.02) [50]. Another study conducted among French population of 38 *MC4R* mutation carriers with obesity and 33 overweight non-carriers found a positive association between the score of disinhibition and *MC4R* mutation (*P* = 0.007), but it was argued that the difference in weight status was not well controlled and could be a potential confounder [51]. Three other studies focusing on TFEQ and polymorphisms near *MC4R* were conducted among French or German population, but no significant results were reported [52–54]. The factor ‘disinhibition’ corresponds the tendency of losing control in ingesting extra quantity of food when exposed to circumstances and cues related with food [55], and the factor ‘hunger’ corresponds to the susceptibility to hunger[49].Although the definitions of the two factors are not exactly the same as food responsiveness and satiety responsiveness in our study, the results in previous studies could explain the appetite and motivational dimension of eating behaviors, which could indirectly confirm our findings.

Different aspects of food intake have been investigated for the association with polymorphisms near *MC4R*. McCaffery, et al. reported that rs571312 was associated with higher caloric intake in white adults (β = 61.32, P = 0.0194) [56]. In 2008, Qi et al. administered a standardized Food Frequency Questionnaire (FFQ) to 4923 overweight women, and reported that rs17782313 was significantly associated with high intakes of total energy (*P* = 0.028), total fat (*P* = 0.008) and protein (*P* = 0.003)[14], while four other studies did not confirm the findings and showed no significant association between polymorphisms near *MC4R* and food intake, either in terms of total energy intake or macro/micronutrient intake, as reviewed by Valette, et al. in 2013 [43]. Another focus is on snacking behaviors. Reported by Stutzmann, et al. [50] in 2009, the *MC4R* rs17782313 C allele showed a significant trend towards higher percentages of snacking, in the population of French obese children (*P* = 0.01), in Swiss obese adults (*P* = 0.04), and in Finnish adolescents as well (*P* = 0.04). We found no published literatures reporting the association of *MC4R* polymorphisms with beverage intake.

Regarding the mediation analysis, our hypothesis was that appetite (i.e. food responsiveness) was the mediator of the SNP association with food intake (i.e. beverage intake). In fact, the other hypothesis that the beverage intake served as the mediator was also possible, but we did not find evidence of population studies supporting the hypothesis. Due to the cross-sectional nature of the current study, the causation of appetite and food intake could not be determined, therefore we could not rule out the possibility that rs12970134 affected appetite through the beverage intake. Also, due to the marginal *P* value of the mediation analysis, both possibilities should be interpreted carefully.

As for the genetic model, we chose to use the additive model in this study. In fact, different genetic models have been used in previous studies, including the dominant model [57], the recessive model [58], and the additive model [59], and there are no conclusions yet which genetic model is the best. However, when investigating at the characterization of the *MC4R* knock-out mice, Cone, et al. found that animals heterozygous for the melanocortin-4 receptor were intermediate to wild type and *MC4R* null mice in obesity-related phenotypes including growth rate, serum leptin concentrations, linear growth, and fasting serum insulin concentrations [45]. The ‘gene dosage effect’ indicated that the risk alleles of polymorphisms within this gene may act in a similar way and follow the additive genetic model.

Several limitations of this study should be noted. First of all, we did not test gene expression or cytokines data of *MC4R*, nor did us test the appetite or food intake in animal models. Further studies were needed for understanding the mechanism of the polymorphisms in/near *MC4R*. Secondly, the food intake in this study was self-reported instead of measured objectively, and we chose several kinds of food intake instead of evaluating food intake systematically. The total caloric intake was not investigated, which could provide more information of the SNP effect on food intake. Thirdly, as children’s eating behaviors were largely influenced by the home environment and their parents’ attitude and behaviors, those information should be investigated in future studies in order to fully assess the genetic effect on eating behaviors. Fourthly, we did not replicate the findings in another independent population, and the sample size of this study was not large. Using the Quanto software, we conducted the power analysis on the beverage intake variable, and the parameters and estimated power were as followings: Using the parent-offspring study design for the continuous variable, with effect allele frequency of 0.2, the Mean±SD of beverage intake of 4.7±6.0 servings/week, and the effect of rs12970134 on beverage intake of 3.8 (unstandardized β), under the additive genetic model, at a two-sided significance level of P<0.05, the analysis showed that 151 trios could reach the power of 90.6%.

Considering the study design in the current study, we could only conclude that there was SNP association with eating behavior in overweight and obese Chinese children population, but whether or not the association existed in the general population could not be decided. Besides, when testing the association between rs12970134 and eating behaviors, 7 behaviors were tested and 4 statistical models were performed for each behavior, which resulted in the multiple-testing problem. Using a *P*<0.0018 Bonferroni criteria, the association between SNP and beverage intake in children was still significant after multiple-testing correction. However, the SNP association with FR or SR had only marginal *P* values, which should be interpreted with caution. In our study, the SNP was not associated with BMI-sds in the children dataset. Since mediation analyses had the prerequisite that the association between the independent variable and the dependent variable should be significant, we could not analyze the mediation effect of eating behavior on the SNP-adiposity association. Further studies could focus on the hypothesis that rs12970134 could possibly increase adiposity through affecting eating behaviors.

In conclusion, we found the polymorphism near *MC4R* was associated with appetite and beverage intake in overweight and obese Chinese children, and appetite could mediate the effect of the polymorphism near *MC4R* on beverage intake. Uncovering the genetic basis for eating behaviors could lead to develop and implement effective interventional strategies early in life, which could help avoid the health risks related to childhood obesity.

## Supporting information

S1 File Dataset of the 151 families with overweight/obese children. (XLS)
